# Feasibility and acceptability of a single-session perioperative acceptance and commitment therapy workshop for preventing chronic postsurgical pain: a single-arm, non-randomized pilot trial

**DOI:** 10.3389/fpain.2025.1558753

**Published:** 2025-04-08

**Authors:** Jolin B. Yamin, Jenna M. Wilson, Bethany D. Pester, Caroline Allen, JiHee Yoon, Marise C. Cornelius, Diya Dharmendran, Kylie Steinhilber, Madelyn Crago, Savannah Kazemipour, Angelina Franqueiro, Delia Fentazi, Kristin L. Schreiber, Kevin E. Vowles, Robert R. Edwards, Robert N. Jamison, Samantha M. Meints

**Affiliations:** ^1^ Department of Anesthesiology, Perioperative and Pain Medicine, Brigham and Women’s Hospital, Boston, MA, United States; ^2^ Harvard Medical School, Boston, MA, United States; ^3^ Department of Anesthesiology and Pain Medicine, University of Washington Medicine, Seattle, WA, United States; ^4^ Department of Psychology, Indiana University, Indianapolis, IN, United States; ^5^ Department of Psychological and Brain Sciences, Texas A&M University, College Station, TX, United States; ^6^ University of Massachusetts Medical School, Worcester, MA, United States; ^7^ Lake Erie College of Osteopathic Medicine, Erie, PA, United States; ^8^ School of Psychology, Queen’s University Belfast, Belfast, United Kingdom

**Keywords:** spine surgery, ACT, acceptance and commitment therapy, chronic pain, brief intervention

## Abstract

**Objective:**

This pilot trial evaluated the feasibility, acceptability, and preliminary effects of a single-session, group-based Acceptance and Commitment Therapy (ACT) intervention for patients undergoing spine surgery (SS) to prevent chronic postsurgical pain (CPSP).

**Methods:**

Forty-five adults (M_age_ = 64 years) scheduled for SS enrolled and were asked to complete baseline questionnaires, and 28 attended a 5 h virtual ACT workshop, which focused on enhancing psychological flexibility and acceptance. Feasibility was assessed by tracking enrollment and attendance, while treatment credibility, expectancy, and helpfulness were evaluated using the Credibility and Expectancy Questionnaire (CEQ) and the Treatment Helpfulness Questionnaire (THQ). Health-related outcomes, including pain severity and interference (Brief Pain Inventory; BPI), anxiety, and depression (PROMIS-29), were measured at baseline, 1-month, 3-months, and 6-months post-surgery.

**Results:**

Of the enrolled participants, 58% attended the workshop, all of whom completed the entire workshop. CEQ and THQ scores indicated high credibility and helpfulness immediately after the intervention and at 1-month post-surgery. Exploratory analyses examining health outcome changes following ACT during the post-surgery period revealed that pain severity and interference, depression and anxiety all decreased over time.

**Discussion:**

These findings suggest that a single-session ACT intervention is feasible and acceptable for patients undergoing SS and may enhance both pain-related functioning and improve psychological outcomes following surgery. Future research should explore the efficacy of this approach in larger, randomized controlled trials to further establish its impact on CPSP prevention.

## Introduction

Chronic back pain is a major public health problem in the U.S., affecting over 20.5 million adults (1). Back pain causes significant disability, disrupts daily functioning, and leads many individuals to seek medical treatment (2, 3). Spine surgery (SS) is an increasingly common medical intervention for individuals with back pain (4). Based on aging population estimates, spine surgeries are forecasted to increase substantially from 2020–2040 (5). Despite pain relief and improved physical function reported after SS, the postoperative course is challenging, and can include persistent pain and opioid use, and delayed functional recovery. Approximately 20% of patients suffer from chronic postsurgical pain (CPSP) (6) and 15%–35% do not experience notable improvements in physical function (7, 8). CPSP is frequently managed with opioid medications (9–12), despite associations between chronic postsurgical opioid use and negative outcomes such as additional surgeries, depression, and prolonged work absence (13, 14).

To improve postoperative recovery, there is growing interest in psychological interventions for postsurgical pain (15–23). However, these interventions often involve up to 12 weekly sessions, which can be burdensome and difficult to complete (24), particularly in the relatively compressed preoperative timeframe leading up to SS (2–6 weeks), among patients with high levels of pain-related interference (25, 26). Accessibility is further limited by provider availability and cost (27). Thus, more accessible and less burdensome psychological interventions are needed. Telehealth offers one solution to these barriers (28, 29). More brief interventions have shown efficacy in pain populations; for example, a single-session cognitive-behavioral treatment is as effective as an eight-session program for pain management (30), and brief Acceptance and Commitment Therapy (ACT) interventions improve pain, function, and psychological health (31, 32). However, research on brief interventions for preventing CPSP remains limited, especially in patients undergoing SS.

ACT is an established approach for chronic pain (33, 34). The primary treatment goal of ACT is to enable people with chronic pain to live meaningful lives with pain. Thus, ACT differs from symptom control, “pain management”, approaches in that the focus is firmly on effective functioning. An ACT intervention is highly applicable in perioperative care because of ACT's focus on helping patients live well with ongoing pain and discomfort and thereby minimize ineffective avoidance behaviors, which are linked to the development of chronic pain (31, 35). By fostering psychological flexibility, ACT can prevent the fear-avoidance cycles that can contribute to the development of CPSP (35–37). Further, ACT has the potential to enhance psychological health and meaningful living outcomes following surgery.

Several studies have examined ACT interventions for managing post-surgical pain and opioid use (20, 38, 39), and additional work has explored the feasibility of brief ACT interventions in perioperative settings (e.g., breast cancer surgery, bariatric surgery, and orthopedic procedures (21, 40, 41). While multiple studies have tested single-session or brief ACT formats in surgical populations, none have specifically examined ACT for patients undergoing spine surgery. The current study builds on this literature by evaluating a single-session perioperative ACT workshop tailored to individuals undergoing spinal surgery, a population at high risk for post-operative chronic pain.

This single-arm, non-randomized pilot trial aimed to evaluate the feasibility and acceptability of a single-session, group-based ACT workshop delivered perioperatively for patients undergoing SS using a mixed method approach. Participants completed baseline questionnaires and were then scheduled to attend a single-session ACT workshop (with between one and four other individuals; attended no more than 60 days prior or within 45 days after their surgery). Immediately following the workshop, participants completed questionnaires assessing the credibility, expectancy, and helpfulness of the intervention and completed qualitative interviews to provide more detailed feedback. Credibility, expectancy, and helpfulness were also assessed 1-month post-surgery. Further, to examine the potential impact of this intervention on health-related outcomes (an exploratory aim), participants completed measures of pain and psychological health outcomes at 1-, 3-, and 6-months post-surgery.

## Materials and methods

### Participants and recruitment

This study was approved by the Mass General Brigham (MGB) Institutional Review Board (protocol # 2022P002302). Study recruitment and data collection were completed between January 2023 and February 2024. A total of 45 participants were enrolled in this pilot trial. Potential participants were identified by the study team and contacted through the patient portal of the electronic medical record system at MGB or via mail. Additionally, providers at MGB (e.g., orthopedic surgeons and neurosurgeons) provided patients with study materials and contact information for the study team. Lastly, we recruited from the community via fliers posted at medical clinics outside the MGB network. Phone calls and REDCap were utilized to screen participants using the inclusion and exclusion criteria. Participants were included in the study if they: (1) were scheduled to undergo a spine surgery (fusion, discectomy, kyphoplasty, or foraminotomy); (2) were adults 22 years or older (42); and (3) were able to communicate fluently in English. Participants were excluded from the study if they: (1) were unable to complete the study procedures due to delirium, dementia, psychosis, or other cognitive impairment; (2) had a history of severe neurological movement disorder; (3) were pregnant or intended to become pregnant during the study (due to impact of sex hormones on pain) (43); (4) had previously undergone spine surgery; (5) had spinal deformity, pseudoarthrosis, trauma, infection, or tumor as the primary indication for surgery; and/or (6) had completed an ACT intervention within the last two years.

### Procedures

**Figure 1:**
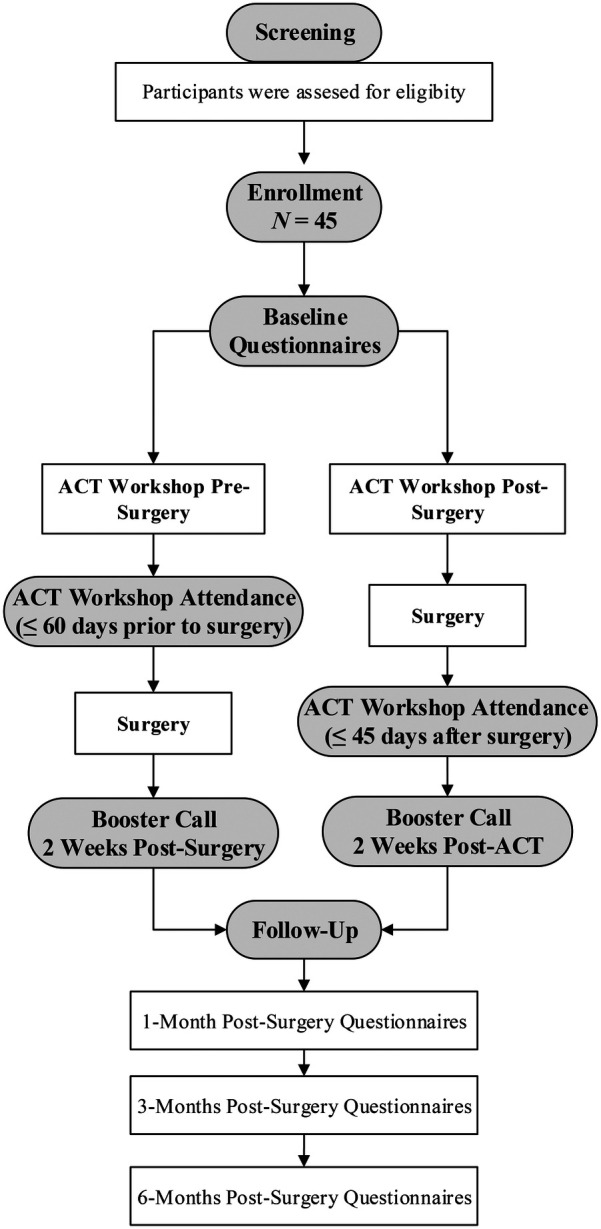
Participant flow through the study.

Following the phone screening, participants completed informed consent, were enrolled in the study, and completed baseline questionnaires of pain and other health-related outcomes. To allow for flexibility with scheduling and patient preferences, participants then attended the group-based ACT workshop no more than 60 days prior *or* within 45 days after their surgery. Immediately following participation in the ACT workshop, participants completed measures assessing treatment credibility, expectancy, and helpfulness, and completed a qualitative interview about the ACT workshop within approximately one week of attending the session. In addition to participating in the workshop, participants received a 30 min booster phone call two weeks post-workshop or post-surgery, whichever was later. During the booster call, ACT principles were reinforced, and participants were given the opportunity to problem-solve with the clinician to address any difficulties with engaging in ACT exercises and practices. Subsequently, participants completed measures of treatment credibility, expectancy, and helpfulness at 1-month post-surgery, and measures of pain and health-related outcomes 1-month, 3-months, and 6-months post-surgery. See Figure 1 for participant flow through the study.

#### Acceptance & commitment therapy (ACT) workshop

The ACT protocol is based on the ACT on Goals Group Program: Acceptance and Commitment Therapy for Chronic Pain and Illness (44). The protocol was designed to help individuals respond to chronic pain through the principles of ACT by enhancing present-focused awareness, increasing willingness skills to decrease ineffective struggles for symptom control, values clarification exercises, and engagement in valued activities with awareness and willingness. The overarching process in ACT, psychological flexibility, represents this present, willing, and flexible approach to living with ongoing, and at times unpredictable, symptoms. The development and refinement of the ACT workshop protocol occurred across 8 treatment groups via clinical observation by the group facilitators, meetings with the primary study team, and based on feedback from qualitative interviews conducted with each study participant within approximately 1-week after their attendance of the workshop (interview questions can be found in Supplementary Appendix A). The study protocol was revised on an iterative basis following each group session. For example, following the first workshop, participants indicated that the metaphor used to introduce the “observer self” concept was difficult to follow. As a result, we excluded the original metaphor (“Sky and Weather” metaphor) to a new metaphor (“CEO” metaphor). Additionally, based on feedback from each session, we minimized introductions within each module to allow for more focus on the experiential exercises and debriefing after them. Although we did receive feedback that participants would prefer two shorter sessions rather than a single longer workshop, when we offered this, only one potential participant expressed interest, and we reverted to the single session format. The finalized ACT intervention was a single-session, 5 h, group workshop comprised of 7 modules and conducted virtually via Zoom. Sessions were conducted monthly with four to seven participants in each group. The seven modules of the protocol were focused on the following topics: (1) Helpful vs. unhelpful solutions for pain, illness, and life; (2) The six core skills of ACT; (3) Introduction to values; (4) Mindfulness; (5) Managing unhelpful thoughts using psychological flexibility skills; (6) Willingness and acceptance; and (7) Putting it all together. Table 1 provides an overview of module content. The intervention sessions were facilitated by at least one clinical psychologist, and participants had the opportunity to share and discuss their experiences with one another.

**Table 1:** Overview of ACT intervention content.

Module	Outline of content
1. Helpful versus unhelpful solutions for pain, illness, and life	• Introductions of facilitators and group members • Introduction to ACT • 5 × 5 × 5 mindfulness exercise • Brief review of mindfulness • Review of history of pain treatments • Solutions exercise and hungry tiger metaphor • 10 min break
2. The six core skills of ACT	• Review of Mindfulness (mindfulness of an object exercise) • Review of the observing self (CEO metaphor) • Review of values (sweet spot exercise) • Review of committed action (rope bridge metaphor) • Review of thought defusion (blind driving metaphor) • Review of acceptance (ball in a pool metaphor) • 10 min break
3. Introduction to values	• Values visualization and my party exercise • Sharing values and noticing discrepancies • Health values, committed action, and S.M.A.R.T. goal setting • 5 min break
4. Mindfulness	• Introduction to mindfulness • Present focus awareness training • 30 min lunch break
5. Overcoming barriers 1: What to do with unhelpful thoughts	• Introduction to thought defusion and distancing • The card exercise • 5 × 5 × 5 mindfulness exercise • Review of thought bombs • Thought defusion techniques (e.g., Titchener's repetition, singing thought out) • 5 min break
6. Overcoming barriers 2: Willingness to experience what cannot be changed	• Introduction to willingness • Unwelcome party guest video • Passengers on a bus video • Acceptance of difficult emotions exercise • Leaves on a stream exercise • Loving kindness exercise • Review of acceptance techniques • 5 min break
7. Putting it all together	• Review of skills • Applying ACT to your life exercise • Wrap up

### Measures

#### Feasibility

Feasibility was assessed by systematically tracking potential participants approached, screened, enrolled in the study, and those who completed and did not complete the ACT workshop. The following specific feasibility metrics were assessed: number of potential participants approached; number screened; number eligible; number enrolled in the study; number who attended workshop; number who completed the booster phone call; and number who completed post-surgery questionnaires.

#### Treatment expectancy and credibility

The Credibility and Expectancy Questionnaire (CEQ) (45) is a measure of treatment expectancy and rationale credibility for use in clinical outcome studies. The CEQ consists of the treatment credibility and treatment expectancy subscales. The treatment credibility subscale consists of three items to indicate the logic, success, and confidence the participant has in the treatment. Scores were summed, with higher scores indicating greater treatment credibility (range: 3–27). The treatment expectancy subscale consists of three items used to assess the likelihood of the success of the treatment to reduce pain and improve function. Scores were summed, where higher scores indicate greater expectancy for beneficial treatment outcomes (range: 1–29). Although there are no validated cut scores for the CEQ, consistent with the scoring anchors used in the measure, we considered credibility scores <9 as not credible, 9–21 as somewhat credible, and scores >21 as very credible. Participants' ratings of expectancy falling below 9 are considered low expectation, 9–21 as moderate expectation, and >21 as high expectations. The CEQ credibility (*α* = .89–.97) and expectancy subscales (*α* = .94–.95) demonstrated excellent reliability in this sample.

#### Treatment helpfulness

The single-item Treatment Helpfulness Questionnaire (THQ) (46) measures perception of the helpfulness of an intervention. Participants were asked to rate the ACT workshop on a scale of −5 (extremely harmful) to 5 (extremely helpful), with 0 indicating “neutral.”

#### Qualitative interview

We conducted 60 min semi-structured remote interviews via telehealth software in the week following the ACT workshop. The goal was to obtain feedback on the ACT workshop. Specifically, we asked participants about the following topics: format (e.g., telehealth, group size, duration); content (e.g., opinions about specific topics and exercise); satisfaction, helpfulness, and suggestions for improvement. Participants provided verbal consent, in addition to written consent, to record these interviews for qualitative analysis. Interviews were conducted by two research assistants, who coded the interview scripts, and provided feedback to the interventionists in the study.

#### Pain severity and interference

The Brief Pain Inventory (BPI) was used to assess for pain severity and pain interference (47). Participants rated the severity of their worst pain, least pain, average pain, and current pain on scales from 0 (no pain) to 10 (pain as bad as you can imagine). These items were averaged to calculate participants' total pain severity score, where greater values represented greater pain severity. Participants then indicated how much pain interfered with their daily functioning including their general activity, mood, walking ability, relationships, ability to conduct normal work, sleep, and ability to take part in leisure activities. Participants rated each variable on a scale from 0 (pain does not interfere) to 10 (pain completely interferes). These variables were then averaged to obtain a total pain interference score where greater values represent greater pain interference. Reliability within this sample for pain severity (*α* = .89–.95) and pain interference (*α* = .94–.97) was excellent.

#### Psychological health

The Patient Reported Outcomes Measurement Information System-29 (PROMIS-29) (48) was utilized to assess participants' anxiety and depression symptoms. Participants were asked to rate how often they experienced each symptom over the previous seven days. The items are rated on a scale of 1 (never) to 5 (always). PROMIS-29 has been validated for use as an outcome measure among populations who receive spine surgery and is responsive to changes in symptoms over time (49, 50). The PROMIS anxiety subscale demonstrated excellent reliability across timepoints in this sample (*α* = .84–.95), similar to previous studies (*α* = .80–.91), and the reliability of the PROMIS depression subscale was poor at 1-month post-surgery (*α* = .57) but was good at the other study timepoints (*α* = .87–.89) which is also consistent with previous studies (*α* = .80–.95) (51).

### Data analysis

Quantitative data were analyzed using IBM SPSS Statistics (V29). To assess feasibility, two-sample *t*- and chi-square tests compared participants who completed the ACT workshop and participants who did not complete the workshop on sociodemographic variables and baseline levels of outcome variables. To assess intervention acceptability, we generated descriptive statistics (e.g., mean, standard deviation) for the CEQ and THQ. We utilized developmental formative evaluation, a type of rapid qualitative analysis (52), to analyze the feedback data from participant interviews following participation in the ACT intervention. The trained research assistant who conducted the interviews independently coded the interview transcripts. Both a deductive (based on our study framework) and an inductive approach to identify any emerging themes not captured by our initial framework were applied. After coding, the assistants compared their findings and reconciled any discrepancies through discussion, thereby refining our coding scheme. Finally, the agreed-upon themes were used to summarize the qualitative data and informed targeted feedback to the interventionists, and changes were made to the ACT protocol on an iterative basis (please see above description of the intervention for examples of changes made based on qualitative feedback). We include a summary of our qualitative findings below. Developmental formative evaluations are ideal for delivering timely results for subsequent intervention development as they allow for modifications to the on an iterative basis which allows for more successful implementation (53–55). To explore the potential impact of the intervention on participants' health outcomes (BPI pain severity and interference, PROMIS anxiety and depression) over time (baseline, 1-month, 3-month, and 6-month post-surgery), we used linear mixed-effect models, which allow for data that are missing at random across participants (56). Because a subset of participants (*N* = 5) completed the ACT workshop after the 1-month post-surgery follow-up, we also conducted sensitivity analyses excluding these participants.

## Results

The sample included 45 participants (*M* age = 64 years, *SD* = 14.5 years; range: 28–85 years). Of the 45 participants, 47% (*n* = 21) were female, 51% (*n* = 23) were male, and 2% did not respond (*n* = 1). Participants were predominantly White (84%; *n* = 37) and non-Hispanic (93%; *n* = 41). Sample demographics are in Table 2.

**Table 2:** Group comparisons on sociodemographic variables and outcome variables at baseline.

Variable	Total Sample (*n* = 44)	Completed ACT workshop (*n* = 26)	Did not complete ACT workshop (*n* = 18)	*t/χ^2^*	*p*
Age (*M, SD*)	64.0 (14.50)	62.75 (13.35)	65.9 (16.35)	2.53	.112
Sex (*n, %*)					
Women	21 (47.7)	15 (57.7)	6 (33.3)		
Men	23 (52.3)	11 (42.3)	12 (66.7)		
Race (*n, %*)				5.03	.284
White/European American	37 (84.1)	22 (84.6)	15 (83.3)		
Black/African American	2 (4.5)	1 (3.8)	1 (5.6)		
Asian	2 (4.5)	2 (7.7)	0 (0)		
Other	1 (2.3)	1 (3.8)	0 (0)		
Prefer not to answer	2 (4.5)	0 (0)	2 (11.1)		
Ethnicity (*n*, *%*)				1.57	.455
Hispanic	2 (4.5)	1 (3.8)	1 (5.6)		
Not Hispanic	41 (93.2)	25 (96.2)	16 (88.9)		
Prefer not to answer	1 (2.3)	0 (0)	1 (5.6)		
Outcome Variables at Baseline (*M*, *SD*)					
BPI pain severity	4.80 (2.06)	4.88 (2.03)	4.66 (2.20)	−.275	.785
BPI pain interference	5.10 (2.87)	4.89 (2.89)	5.48 (2.93)	.546	.589
PROMIS anxiety	8.38 (3.65)	8.20 (3.78)	8.64 (3.59)	.343	.734
PROMIS depression	7.50 (3.37)	6.90 (2.77)	8.36 (4.03)	1.25	.220
Survey Completion (*n,%*)					
Baseline	44 (100)	26 (100)	18 (100)	1.40	.422
1-Month	32 (73)	24 (92)	8 (44)	13.47	<.001
3-Month	29 (66)	23 (88)	6 (33)	15.50	<.001
6-Month	30 (68)	24 (92)	6 (33)	18.22	<.001

### Feasibility and acceptability

A total of 353 patients were approached for the study, of whom 98 (28%) were screened, and 75 were eligible to participate. Participants were excluded due to age (*N* = 1), severe cognitive impairment (*N* = 2), and for having a previous spine surgery (*N* = 20). Of the 75 eligible patients, 45 (60%) participants enrolled in the study, 26 (58%) attended the ACT workshop, and 18 (69%) of those who attended the workshop attended the booster call. Of the 45 participants enrolled in the study, 44 (98%) completed baseline measures, 32 (69%) completed 1-month, 29 (64%) completed 3-month, and 30 (67%) completed 6-month post-surgery questionnaires. The t- and chi-square tests comparing participants who attended the ACT workshop to those who did not attend the workshop on demographics and baseline levels of all health outcomes revealed no significant between-group differences in any of the variables (See Table 2). However, there were significant differences in drop-out rates at each post-surgery follow-up time point, where those who attended the ACT workshop were more likely to complete post-surgery questionnaires at each time point (*p*s < .001). Of the 26 participants who attended the ACT workshop, 10 attended before surgery, and 16 attended after surgery.

Participants rated the ACT workshop as somewhat to very credible (credibility) and had moderate expectations for it to be successful in reducing pain and improving function (expectancy) both immediately following the intervention (credibility *M* = 20.83, *SD* = 5.37 and expectancy *M* = 17.92, *SD* = 7.42, respectively) and at 1-month post-surgery (credibility *M* = 21.05, *SD* = 6.42 and expectancy *M* = 18.85, *SD* = 8.27). Similarly, participants rated the workshop as helpful immediately post-workshop (M = 3.83, SD = 1.40) and at 1-month post-surgery (M = 2.86, SD = 2.50) with 17 (71%) and 12 (55%) participants rating it at or above the median (Mdn = 4) at each time point, respectively. None of the participants who attended the ACT workshop rated it as unhelpful (*x* < 0) immediately following the workshop and only one participant rated it negatively (*x* = −5) at 1-month post-surgery.

### Qualitative feedback from participant interviews

Findings from participants' interviews revealed largely positive feedback on the ACT workshop. Participants mentioned that having the workshop close to the time of their surgery was ideal because they learned coping skills to utilize during the recovery period. Participants also appreciated the virtual format for its convenience, with some participants mentioning that they likely would not have attended the workshop if it required in-person attendance due to barriers related to transportation. Small group sizes were valued for fostering discussion and providing a sense of community and support from other individuals going through a similar situation. However, opinions were mixed on the length of the workshop—while some participants found 5 h manageable and mentioned that they likely would not have attended the workshop if it required several sessions, other participants suggested either shortening the workshop or splitting it into multiple sessions. In terms of content, participants emphasized how they felt that the techniques were helpful, particularly cognitive defusion and mindfulness exercises (5 × 5 × 5 and “leaves on a stream”), such that these techniques taught them how to acknowledge and then accept negative thoughts. Participants also mentioned that it was beneficial to hear instructors apply the techniques to their own lives when providing examples of the applicability of the techniques to real-life situations. Suggestions for improvement of the workshop included allowing more time for practicing the exercises and providing clearer instructions on how to specifically apply the techniques during increased pain episodes.

### Pain outcomes

**Figure 2:**
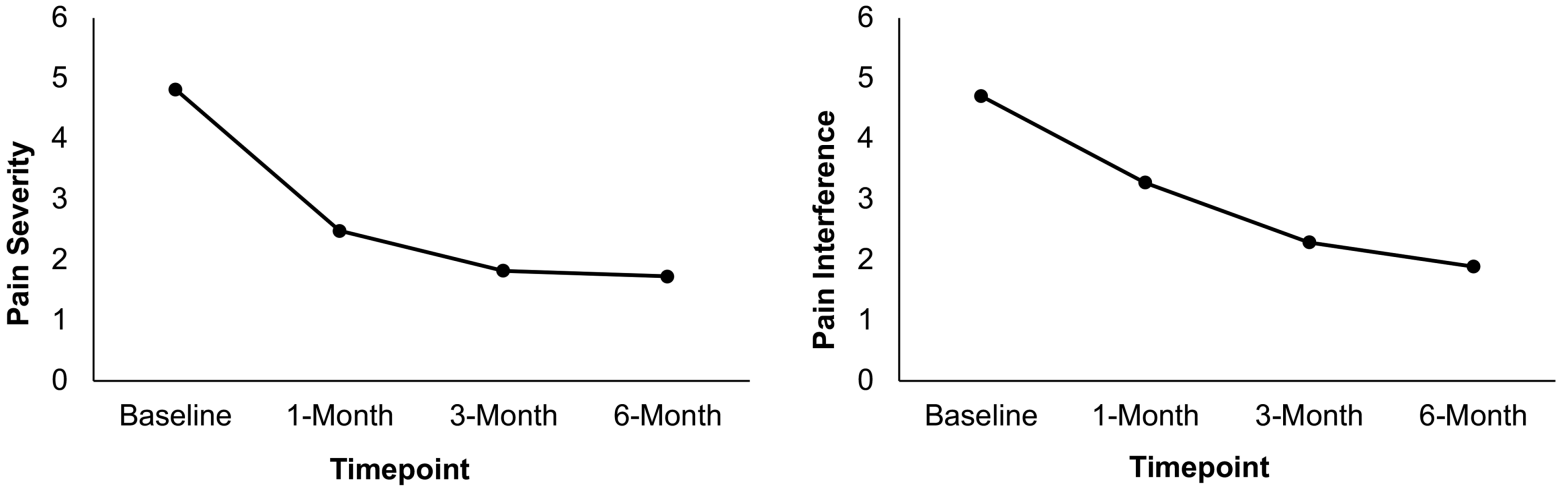
Mean pain severity and interference ratings at baseline, 1-month, 3-month, and 6-month post-surgery in patients who attended the ACT workshop (*N* = 26). Scores are presented using a restricted range for readability; however, PROMIS measures were scored using summed raw scores, with the full scale range available in the PROMIS scoring manual.

Results from a linear mixed model indicated that BPI pain severity differed statistically significantly between time points [*F*(3, 59) = 16.75, *p* < .001]. Pairwise comparisons between baseline and each post-surgery timepoint revealed that BPI pain severity significantly decreased from baseline (*M* = 4.82) to 1 month (*M* = 2.48, *p* < .001), 3 months (*M* = 1.82, *p* < .001), and 6 months (*M* = 1.73, *p* < .001) post-surgery. Similarly, results from a linear mixed model indicated that BPI pain interference differed statistically significantly between time points [*F*(3, 57) = 5.30, *p* = .003]. Pairwise comparisons between baseline and each post-surgery timepoint revealed that BPI pain interference significantly decreased from baseline (*M* = 4.71) to 1 month (*M* = 3.28, *p* = .015), 3 months (*M* = 2.29, *p* < .001), and 6 months (*M* = 1.89, *p* < .001) post-surgery. See Table 3 and Figure 2. Results of sensitivity analyses excluding participants who did not complete the ACT workshop prior to the 1-month post-surgery follow up were consistent with the above findings, indicating that there was a significant decrease from baseline to each post-surgery follow-up timepoint for pain severity (*p*s < .001) and from baseline to 3-month and 6-month post-surgery for pain interference (*p*s < .01) though not between baseline and 1-month post-surgery (*p* = .194).

**Table 3:** Changes in health outcome from baseline to 1-, 3-, and 6-months post-surgery in participants who completed the ACT workshop (*N* = 26).

Outcome	Mean improvement over time	SE	*p*
Pain severity			
Baseline to 1-month	−2.34	0.40	**<**.**001**
Baseline to 3-month	−3.00	0.47	**<**.**001**
Baseline to 6-month	−3.09	0.51	**<**.**001**
Pain interference			
Baseline to 1-month	−1.43	0.57	.**015**
Baseline to 3-month	−2.42	0.69	**<**.**001**
Baseline to 6-month	−2.82	0.74	**<**.**001**
Anxiety			
Baseline to 1-month	−2.53	0.70	**<**.**001**
Baseline to 3-month	−1.74	0.82	.**036**
Baseline to 6-month	−2.30	0.85	.**008**
Depression			
Baseline to 1-month	−1.52	0.47	.**002**
Baseline to 3-month	−1.61	0.57	.**006**
Baseline to 6-month	−1.52	0.62	.**016**

Note. All tests were two-tailed. The *p*-values are bold when they are less than the significance level cut-off of .05.

### Psychological health outcomes

**Figure 3:**
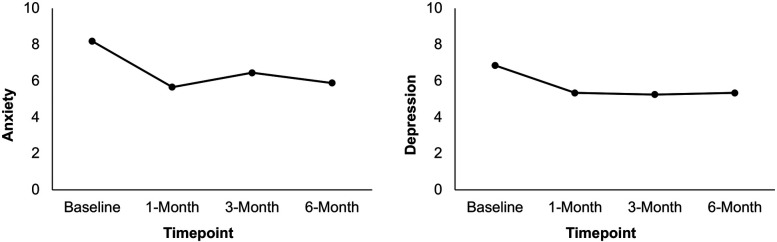
Mean anxiety and depression ratings at baseline, 1-month, 3-month, and 6-month post-surgery in patients who attended the ACT workshop (*N* = 26). Scores are presented using a restricted range for readability; however, PROMIS measures were scored using summed raw scores, with the full scale range available in the PROMIS scoring manual.

Results from a linear mixed model indicated that PROMIS anxiety symptoms differed statistically significantly between time points [*F*(3, 59) = 4.96, *p* = .004]. Pairwise comparisons between baseline and each post-surgery timepoint revealed that anxiety symptoms significantly decreased from baseline (*M* = 8.19) to 1 month (*M* = 5.66, *p* < .001), 3 months (*M* = 6.45, *p* = .036), and 6 months (*M* = 5.89, *p* = .008) post-surgery. Finally, results from a linear mixed model indicated that PROMIS depression symptoms differed statistically significantly between time points [*F*(3, 58) = 3.83, *p* = .014]. Pairwise comparisons between baseline and each post-surgery timepoint revealed that depression symptoms significantly decreased from baseline (*M* = 6.86) to 1 month (*M* = 5.34, *p* = .002), 3 months (*M* = 5.25, *p* = .006), and 6 months (*M* = 5.34, *p* = .016) post-surgery. See Table 3 and Figure 3. Results of sensitivity analyses excluding participants who did not complete the ACT workshop prior to the 1-month follow-up showed a similar pattern of results though failed to meet statistical significance at some time points. While there was a significant decrease in anxiety from baseline to 1-month and 6-months post-surgery (*p*s < .05), the decrease from baseline to 3-months post-surgery failed to reach significance (*p* *=* .277). Likewise, while there was a significant improvement from baseline to 1-month post-surgery in depressive symptoms (*p* *=* .022), reductions between baseline and 3-months and 6-months post-surgery were not significant (*p* = .144; *p* = .129, respectively).

## Discussion

This pilot study explored the feasibility, acceptability, and preliminary effects of a single-session, group-based ACT intervention for patients undergoing SS. Overall, the findings suggest that a brief ACT workshop was a feasible and well-accepted intervention within this patient population, with promising effects on enhancing pain-related functioning and psychological health.

The primary objective of this study was to assess the feasibility and acceptability of delivering the perioperative ACT intervention. While this is a small study, we were able to reach our recruitment target of 45 participants within 13 months. Brigham and Women's Hospital performs approximately 400 spine surgeries annually, many of which are revision surgeries. Thus, our ability to recruit nearly 10% of this population was consistent with our goal. That said, in order to successfully recruit for a larger trial, it would be beneficial to expand enrollment beyond one single hospital. Of the 45 participants enrolled in the study, 58% attended the workshop, with no significant demographic or baseline differences between those who attended and those who did not. This attendance rate, while moderate, indicates that the intervention was generally accessible and acceptable to the sample. In fact, because participants who did not complete the ACT intervention were also more likely to not complete questionnaires post-surgery, it is likely that it was not the intervention itself but instead other factors that resulted in this moderate attendance rate. While some attendees addressed this in their qualitative feedback by suggesting splitting the workshop into two sessions, when we offered this option, participants chose to complete the workshop in a single session rather than splitting into two sessions. Moreover, we do not have qualitative feedback from those who did not attend the workshop or chose not to participate thus we cannot speculate further. Participants provided high ratings for treatment credibility and expectancy, both immediately post-intervention and at 1-month post-surgery, suggesting that the ACT intervention was perceived as logical and beneficial. Similarly, participants rated the intervention as helpful at both time points, although the mean helpfulness scores declined somewhat by 1-month post-surgery. These findings align with previous studies demonstrating that ACT is a credible and acceptable approach for pain treatment in clinical settings (20, 57).

In terms of health outcomes, participants experienced significant reductions in pain severity, pain interference, anxiety, and depression across the 6-month post-surgery period. Pain severity and interference decreased substantially from baseline to 1-month post-surgery and continued to improve at 3- and 6-month post-surgery. While these improvements may reflect the natural course of post-surgical recovery, they are consistent with prior research suggesting that ACT may be beneficial for pain management (18). Additionally, reductions in anxiety and depression suggest potential psychological benefits; however, given the study design, we cannot determine whether these changes are attributable to ACT specifically. These findings support the feasibility of delivering ACT in perioperative settings and highlight the need for a larger randomized trial to evaluate its impact on pain and psychological outcomes in patients undergoing SS (20, 39).

Although ACT has been shown to be effective for chronic pain (57, 58), this study stands out in its focus on a single-session, accessible ACT intervention delivered perioperatively**,** specifically targeting the prevention of chronic postsurgical pain. The inclusion of a booster call post-surgery is another notable feature, designed to reinforce ACT principles and provide ongoing support during the critical recovery period. Additionally, the iterative, patient-centered development of the ACT protocol**,** where feedback from participants was used to modify and refine the workshop content after each group session, is a key strength. This process allowed for real-time adjustments to the intervention, ensuring that it was responsive to the needs and preferences of the target patient population (59, 60). The flexibility of delivering the intervention virtually via Zoom also highlights the study's relevance in the context of modern healthcare delivery, potentially increasing accessibility for patients who face mobility or logistical challenges. This study offers an exploration of how brief, accessible psychological interventions like ACT can be tailored to fit into surgical care pathways, offering valuable insights for future research and clinical practice.

### Limitations and future directions

This study has several limitations. First, the study used a single-arm, non-randomized design, which limits the ability to draw causal conclusions about the efficacy of the intervention. Without a control group, it is difficult to determine whether the observed improvements in pain and psychological health were due to the ACT workshop or to other factors, such as receiving SS, or the natural recovery process following surgery. Future studies should include a randomized controlled trial design to assess the intervention's efficacy more rigorously. Second, while qualitative feedback was generally positive, participants expressed mixed opinions on the length and timing of the workshop. Some participants found the 5 h session too long and suggested splitting it into shorter sessions, while others preferred receiving the intervention either before or after surgery. These insights highlight the need for further refinement of the intervention to enhance its accessibility and engagement. Third, the sample size was small, and the majority of participants were White and non-Hispanic, which limits the generalizability of the findings to more diverse populations. Future research should aim to recruit a larger and more diverse sample to ensure the intervention's applicability across different demographic groups. Lastly, there was a small subset (*N* = 5) participants who completed the ACT workshop following the 1-month post-surgery follow up. As a result, their 1-month post-surgery data did not reflect results from ACT. However, our sensitivity analyses showed a similar pattern of results when these individuals were excluded. Future studies should ensure that the intervention occurs prior to any follow-up assessments.

## Conclusions

This pilot study provides preliminary evidence that a brief, group-based ACT intervention is feasible, acceptable, and potentially effective for preventing chronic post-surgical pain and improving psychological health in patients undergoing SS. The findings underscore the importance of continuing to explore the role of psychological interventions, particularly ACT, in perioperative care. Future research should focus on optimizing the intervention format, incorporating feedback from participants, and conducting larger, randomized trials to confirm these preliminary results. With further refinement, single-session ACT interventions have the potential to become a valuable tool for improving postoperative recovery and preventing chronic pain in this patient population.

## Data Availability

The raw data supporting the conclusions of this article will be made available by the authors, without undue reservation.
