# Supplementary material for: Feasibility and acceptability of a single-session perioperative acceptance and commitment therapy workshop for preventing chronic postsurgical pain: a single-arm, non-randomized pilot trial
Source: Front Pain Res (Lausanne). 2025 Apr 8;6:1558753. doi: 10.3389/fpain.2025.1558753 (PMC12011729; doi:10.3389/fpain.2025.1558753)
Supplement: Supplementary file 1 [file Datasheet1.docx]

**PREPS Qualitative Interview Questions**

*Pre-surgery Interview (~1 week post-workshop)*

## Before we begin, do I have your verbal permission to record our conversation? I would like to repeat that there is no identifiable information in this recording, and it will be labeled only with a participant ID number, not your name. Do you have any questions before we begin the interview? Okay, great. As we begin, remember that there are no “right or wrong” answers, and all of the information you share with us will be kept confidential and will not be shared with your healthcare provider.

We are interested in talking to you about your experience in the ACT workshop you attended. It is important to us to use your feedback to continue making this workshop better for patients so please be honest and let us know anything you think might help us make it better!

1. How are you feeling about your surgery?

I’d like to talk to you about your experience with the workshop you attended.

1. Overall, what did you think about the workshop?
2. How would you describe the workshop to a friend or family?
3. Before attending this workshop, what did you expect to learn or get from it?
   1. How did the workshop differ from what you expected?
4. What new information, perspectives, and/or skills did you take away from this workshop?
5. Are there any skills that you learned that you’ve been using since the workshop?
6. How has what you learned helped you?
7. How have your values informed your decisions since being in the group?
8. How did you feel about what was covered in the workshop?
   1. Was there anything you found particularly useful or impactful? What was the most useful part of the workshop?
      1. What parts of the workshop felt most beneficial as you move forward/that you think you’ll use most in your life? (ensure they give at least one specific part)
   2. Was anything unnecessary or unhelpful? What was the least useful part of the workshop? (again, make sure they provide at least one thing that was least helpful/useful)
   3. Now I’m going to remind you of some specific parts of the workshop. I’d like you to let me your thoughts for each. Was it helpful? Would you add or remove anything? Change anything?
      1. 5X5X5 exercise
      2. Helpful vs. unhelpful solutions for pain, illness, and life (this is where we discussed things you were already doing to try to reduce pain, and the short- and long-term effects of those things)
      3. The 6 core skills of ACT (this was an overview of the 6 core skills, with exercises to briefly practice each one)
         1. Mindfulness of an object
         2. CEO metaphor
         3. Sweet spot memory
         4. Rope bridge metaphor
         5. Blind driving metaphor
         6. Ball in the pool metaphor
      4. Introduction to values
         1. My party exercise
         2. SMART goals
      5. Mindfulness
         1. past-present-future mindfulness exercise
         2. Card exercise
      6. Thought Bombs & Diffusion
         1. Lemon exercise
         2. Sing it out
      7. Willingness
         1. Unwelcome party guest
         2. Acceptance exercise
         3. Leaves on a stream
   4. Was anything missing? Are there any skills or techniques that you expected to learn about or didn’t learn about, but wish you did?
9. How did this workshop impact the way you feel about yourself, your life, and your pain and/or back condition?
10. How satisfied are you with the material covered in the workshop?
11. How did you feel about the format of this workshop?
    1. In-person vs remote vs hybrid
    2. Length (5 hrs)
       1. Would you have preferred this in 2, 3, or 4 sessions instead?
    3. Single session
    4. Group vs individual
    5. Group size
    6. Number of moderators (i.e., group leaders)
    7. Amount of group interaction
12. Did anything surprise you about the workshop?
13. How can we improve the workshop?
14. Is there anything else you would like to add?

*Post-booster Interview*

## Before we begin, do I have your verbal permission to record our conversation? I would like to repeat that there is no identifiable information in this recording, and it will be labeled only with a participant ID number, not your name. Do you have any questions before we begin the interview? Okay, great. As we begin, remember that there are no “right or wrong” answers, and all of the information you share with us will be kept confidential and will not be shared with your healthcare provider.

## Today, I’m interested in hearing how your surgery went and to learn more about your experience in the ACT workshop you attended before surgery.

1. How did your surgery go?
2. What has your recovery been like?

I want to revisit the workshop you did and talk a little bit about that.

1. Now that it’s been a little while, what do you think about the workshop?
2. What information, concepts, or skills do you remember learning at the workshop?
   1. Which of these concepts or skills have you been using in your daily life? What skills have helped you cope or manage your pain?
      1. What has your experience been like using these skills (is it easy, difficult)?
   2. What barriers have come up that have made it difficult to apply these teachings and skills?
   3. What has been most useful for your life? For your pain? What was the most useful part of the workshop? What did you like?
   4. Do you recall any material or exercises that were unhelpful or unnecessary? What was the least useful part of the workshop? What did you not like, or wish was done differently?
3. What would have been helpful to learn or discuss at the pre-surgical workshop that was not covered?
4. Did you find it helpful to have a booster session?
   1. Would you change the timing or format of the booster session?
5. Would you recommend the workshop to a friend/family?
   1. If no, What would change your mind?
6. Is there anything else you would like to add?
